# Distribution of periodontopathic bacterial species in Japanese children with developmental disabilities

**DOI:** 10.1186/1472-6831-9-24

**Published:** 2009-09-23

**Authors:** Shuhei Naka, Aki Yamana, Kazuhiko Nakano, Rena Okawa, Kazuyo Fujita, Ayuchi Kojima, Hirotoshi Nemoto, Ryota Nomura, Michiyo Matsumoto, Takashi Ooshima

**Affiliations:** 1 Department of Pediatric Dentistry, Osaka University Graduate School of Dentistry, Suita, Osaka, Japan

## Abstract

**Background:**

Recent developments in molecular biological techniques have enabled rapid detection of periodontopathic bacterial species in clinical specimens. Accumulated evidence suggests that detection of specific bacterial species enables identification of subjects at high risk for the onset of periodontitis. We investigated the distribution of 10 selected periodontopathic bacterial species in dental plaque specimens obtained from children with disabilities who were attending daycare centers.

**Methods:**

A total of 187 children (136 boys, 51 girls) aged 1-6 years old and diagnosed with such disabilities as mental retardation, cerebral palsy, and autism, participated in the study. Subgingival dental plaque specimens were collected from the buccal side of the maxillary left second primary molar after a clinical examination. Bacterial DNA was extracted from the specimens and PCR analyses were carried out to detect 10 selected periodontopathic species using specific primers for each. In addition, statistical analyses were performed to analyze the correlations among clinical parameters and the detected species.

**Results:**

The most frequently detected species was *Capnocytophaga sputigena *(28.3%), followed by *Aggregatibacter actinomycetemcomitans *(20.9%) and *Campylobacter rectus *(18.2%). *Eikenella corrodens*, *Capnocytophaga ochracea*, and *Prevotella nigrescence *were detected in approximately 10% of the specimens, whereas *Treponema denticola*, *Tannerella forsythia*, and *Prevotella intermedia *were rarely found, and *Porphyromonas gingivalis *was not detected in any of the subjects. The total numbers of detected species were positively correlated with the age of the subjects. There were 10 subjects with positive reactions for *T. denticola *and/or *T. forsythia*, in whom the total number of bacterial species was significantly higher as compared to the other subjects. Furthermore, subjects possessing *C. rectus *showed significantly greater values for periodontal pocket depth, gingival index, and total number of species.

**Conclusion:**

We found that approximately one-fourth of the present subjects with disabilities who possessed at least one of *T. denticola*, *T. forsythia*, and *C. rectus *were at possible risk for periodontitis. Follow-up examinations as well as preventive approaches should be utilized for such individuals.

## Background

Gingivitis is the most common periodontal disease among children and its primary etiological factor is considered to be bacterial plaque [1]. Studies of bacterial profiles in dental plaque specimens have been performed worldwide [2-6]. In previous studies of Japanese children, we investigated the presence of the following 10 periodontal species considered to be associated with periodontal diseases, based on other reports: *Porphyromonas gingivalis *(Pg), *Tannerella forsythia *(Tf), *Prevotella intermedia *(Pi), *Prevotella nigrescence *(Pn), *Campylobacter rectus *(Cr), *Eikenella corrodens *(Ec), *Aggregatibacter actinomycetemcomitans *(Aa), *Capnocytophaga ochracea *(Co), *Capnocytophaga sputigena *(Cs) and *Treponema denticola *(Td) [7-9]. Those studies revealed that approximately 15% of periodontally healthy subjects were positive for none of those 10 species, with Cr, Ec, Aa, Cs, and Co found in half of the dental plaque specimens, and the detection rates of Tf, Pi, Pg, and Td extremely low [7,8]. In addition, the average number of those 10 species was shown to gradually increase from 2.17 species for subjects at the age of 2 years to 3.00 species for those at the age of 6 [7].

Accumulation of data from various studies has revealed which species are virulent for periodontitis, with possession of Pg, Tf, and Cr reported to indicate risk for the onset of periodontal diseases in Japanese subjects [10,11]. In addition, the red complex species, Pg, Td, and Tf, are known to be causative agents of periodontitis and highly associated with its severity [12,13]. Furthermore, children positive for these species have been shown to be at risk for infection by a high number of species during the adolescent period [14]. On the other hand, subjects possessing Cr were reported to also possess the red complex species at a significantly higher rate as compared to those without Cr [15]. The association of Down's syndrome with early onset of periodontitis is well known and various periodontopathic species have been reported to be colonized during very early childhood, among which Tf, Td, Pn, Cr, and Pg were each shown to have a significantly high detection frequency as compared to age-matched healthy children [16]. On the other hand, there are few reports describing the distribution of periodontopathic species in children with developmental disabilities. In the present study, we investigated bacterial profiles of dental plaque specimens collected from subjects with various developmental disabilities who attended the daycare centers.

## Methods

### Subjects and clinical specimens

The study protocol was approved by the Ethics Committee of Osaka University Graduate School of Dentistry (No. H19-E10). The guardians of the subjects were informed of the contents of the study and approved their participation. We studied 187 children (136 boys, 51 girls; age range 1 to 6 years old), from whom dental plaque specimens were collected in June 2009. The subjects were attending daycare centers located in the southern part of Osaka for children with developmental disabilities, such as mental retardation (MR), cerebral palsy (CP), autism (AU), and pervasive developmental disorders (PDD). Diagnoses of those developmental disabilities in each individual were performed by pediatricians, who were asked by the daycare center staff to perform examinations when the guardians applied for the children to attend the centers. Oral hygiene procedures, such as brushing, were performed by their guardians at home as well as the daycare center staff when the subjects attended the centers. This is the second year for us to perform annual dental examinations at these subjects in the daycare centers.

Subgingival dental plaque specimens were collected according to a method described previously [7,8,14], with some modifications. After brushing instruction was given to and performed by the guardians for removal of supragingival food debris, subgingival dental plaque specimens were collected from the buccal side of the maxillary left second primary molar in subjects aged 3 years old and more. For younger subjects, the collection area was the most distal completely erupted tooth in the maxillary left quadrant, which was chosen, to avoid the physiological effects on unstable periodontal conditions when evaluating the subject. In addition, all subjects in the present study had only primary dentition, and those with mixed dentition were excluded. All dental plaque specimens were obtained with sterile Gracey curettes and suspended in sterile phosphate-buffered saline on ice, then transported to our laboratory for extraction of bacterial genomic DNA.

### Clinical examinations

All clinical examinations were carried out by a single skilled examiner (MM). Clinical parameters, including probing depth of the periodontal pocket (PD), bleeding on probing (BOP), plaque index (PI) [17], and gingival index (GI) [18], were recoded for the teeth from which dental plaque specimens were collected. PD was measured to the nearest millimeter at 6 points around the circumference of each tooth (mesio-, mid-, and disto-buccal; and disto-, mid-, and mesio-lingual) from the gingival margin to the deepest probing point, using a round-ended probe tip 0.4 mm in diameter. BOP was scored as (+) for immediate bleeding on probing or (-) for no bleeding.

### Identification of periodontopathic bacterial species

Microbiological analyses were performed using methods described previously [7-9,14]. Briefly, the specimens in sterile saline were centrifuged at 15000 rpm for 5 min to pellet the bacterial cells, then bacterial genomic DNA was extracted from each pellet using a DNA isolation kit (Puregene, Gentra Systems, Minneapolis, MN, USA). The presence of 10 periodontitis-associated bacterial species, Pg, Tf, Pi, Pn, Cr, Ec, Aa, Co, Cs, and Td, was then determined in the specimens by PCR using species-specific sets of primers [see Additional file 1] [2-6]. Specificity with this method was shown in those studies, with the minimum detection level for these species reported to range from 10-100 cells.

### Statistical analyses

Statistical analyses were performed using the computational software packages StatView 5.0 (SAS Institute Inc., Cary, NC, USA) and Prism 4 (GraphPad Software Inc., San Diego, CA, USA). The total numbers of bacterial species by age group (1-2, 3, 4, and 5-6 years old) were analyzed using ANOVA (Tukey's Multiple Comparison Test) and the correlations of those values to clinical factors were evaluated by regression analysis. Clinical parameters and the total number of bacterial species in subjects with or without red complex species, and with or without Cr were analyzed by Student's t-test. Also, the clinical parameters, PD, PI, and GI were compared with the total number of bacterial species using ANOVA. In addition, BOP and the percentage of subjects positive for red complex species, as well as the distribution rates of red complex species and Cr were analyzed with Fisher's exact probability test. *P *values less than 0.05 were considered to be significant.

## Results

### Prevalence of each periodontopathic bacterial species

**Figure 1:**
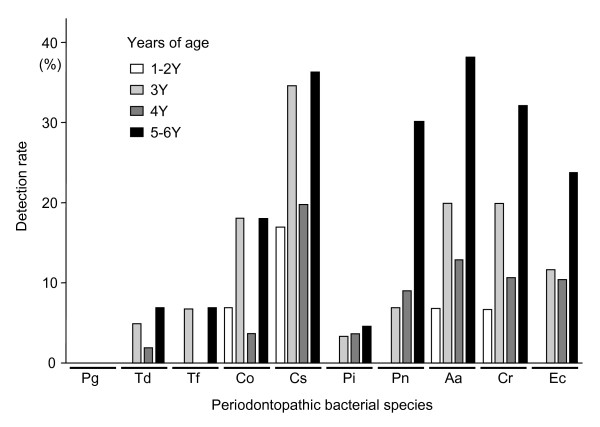
**Frequency and distribution of 10 periodontopathic bacteria by age group**. Pg, *Porphyromonas gingivalis*; Td, *Treponema denticola*; Tf, *Tannerella forsythia*; Co, *Capnocytophaga ochracea*; Cs, *Capnocytophaga sputigena*; Pi, *Prevotella intermedia*; Pn, *Prevotella nigrescence*; Aa, *Aggregatibacter actinomycetemcomitans*; Cr, *Campylobacter rectus*; Ec, *Eikenella corrodens*.

The most frequently detected species was Cs (28.3%), followed by Aa (20.9%), Cr (18.2%), Ec (12.8%), Co (12.3%), and Pn (11.8%) (Figure 1). As for red complex species, the detection rates were comparatively quite low, with a prevalence of 3.7% for Td and Tf, while that of Pi was 3.2%. In addition, Pg was not detected in any of the 187 subjects. The detection rate for each species showed an increasing trend with age.

### Total number of the periodontopathic bacterial species

**Figure 2:**
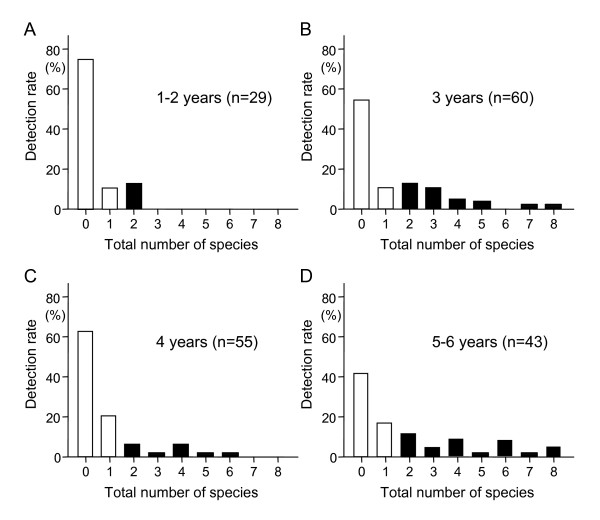
**Percentage of subjects with 0 to 8 of the target bacterial species by age group**. The subjects were divided into 4 groups based on age, as follows: 1-2 (A), 3 (B), 4 (C), and 5-6 (D) years old. Open and closed rectangles indicate subjects with low and high risk, respectively, for infection by the red complex species in the future based on findings in our previous study.

The mean value for the total number of the 10 tested bacterial species in all subjects was 1.16 species, while the group of subjects age 5-6 years old showed the highest mean value of 1.98 species (Table 1). There were no significant differences for the clinical parameters PD, PI, GI, and BOP among the age groups. On the other hand, the total number of bacterial species was positively correlated with age (*P *= 0.0057), while PD, PI, and GI tended to be positively correlated with age, though it was not significant (Table 2). The rate of subjects in the group aged 1-2 years old with no species detected was 75.9% and that rate decreased with age (3 years old, 55.0%; 4 years old, 63.6%; 5-6 years old, 41.9%) (Figure 2). Approximately 60% of the subjects in each age group were found to possess 0 or 1 species. In addition, the number of subjects with a greater number of species increased with age, with a maximum of 8 species identified in a subject.

**Table 1:** Results of clinical and molecular biological examinations of 187 subjects

**Age**	**Number of subjects**			**Clinical parameters^a^**				**PCR results**	
			
	**Boys**	**Girls**	**Total**	**PD** **[Mean ± SE (mm)]**	**PI** **[Mean ± SE]**	**GI** **[Mean ± SE]**	**BOP** **-positive** **(%)**	**Total number of 10 species [Mean ± SE]**	**Subjects positive for red-complex species (%)**
1-2	18	11	29	2.07 ± 0.14	1.61 ± 0.23	0.48 ± 0.13	10.3	0.38 ± 0.14^b^	0
3	46	14	60	2.30 ± 0.09	1.78 ± 0.13	0.40 ± 0.09	8.3	1.28 ± 0.24	8.3
4	39	16	55	2.33 ± 0.08	1.49 ± 0.15	0.33 ± 0.07	3.6	0.78 ± 0.19^c^	1.8
5-6	33	10	43	2.58 ± 0.10	1.56 ± 0.17	0.35 ± 0.09	9.3	1.98 ± 0.37^b, c^	9.3

Total	136	51	187	2.34 ± 0.05	1.62 ± 0.08	0.38 ± 0.05	7.5	1.16 ± 0.13	5.3

^a^PD; periodontal pocket depth, PI; plaque index, GI; gingival index, BOP; bleeding on probing.

^b, c^Significant differences revealed by ANOVA (Tukey's Multiple Comparison test) (*P *< 0.01).

**Table 2:** Clinical factors correlated with total number of detected bacteria determined by regression analysis

	**Values**			**Statistical results**	
		
**Clinical factors**	**Min.**	**Max.**	**Median**	***P *values**	***r *values**
Age	1.08	6.08	4.08	0.0057	0.20
Periodontal pocket depth (PD) (mm)	1	4	2	0.0560	0.14
Plaque index (PI)	0	3	2	0.0637	0.14
Gingival index (GI)	0	3	0	0.0969	0.12

### Clinical and molecular biological analyses of subjects positive or negative for red complex species or Campylobacter rectus (Cr)

There were 10 subjects with positive reactions for Td and/or Tf (5.3%), who were classified as the red complex positive [RC (+)] group. On the other hand, no significant differences in regard to the values for PD, PI, GI, and BOP-positive rate between the RC (+) group and subjects in the RC (-) group were identified (Table 3). In contrast, the total number of the 10 bacterial species in the RC (+) group was 6.00, which was significantly higher than that in the RC (-) group (*P *< 0.001). As for the subjects positive for Cr, red complex species were detected in 8, whereas the other 26 subjects were negative for red complex species. PD and GI values, as well as the total number of bacterial species in subjects positive for Cr [Cr (+) group] were significantly greater than in the Cr (-) group. In addition, the values for PI and BOP-positive rate in the Cr (+) group were higher than those in the Cr (-) group, though the difference was not significant.

**Table 3:** Clinical and molecular biological examination results for subjects positive or negative for red complex (RC) species or *Campylobacter rectus *(Cr)

**Groups**	**Clinical parameters^a^**				**PCR results**
		
	**PD** **[Mean ± SE (mm)]**	**PI** **[Mean ± SE]**	**GI** **[Mean ± SE]**	**BOP** **-positive** **(%)**	**Total number of species [Mean ± SE]**
RC (+) (n = 10)	2.70 ± 0.15	1.60 ± 0.40	0.40 ± 0.22	10.0	6.00 ± 0.67^d^
RC (-) (n = 177)	2.32 ± 0.05	1.62 ± 0.08	0.38 ± 0.05	7.4	0.88 ± 0.11^d^

Cr (+) (n = 34)	2.56 ± 0.11^b^	1.91 ± 0.19	0.62 ± 0.13^c^	14.7	4.18 ± 0.34^e^
Cr (-) (n = 153)	2.28 ± 0.05^b^	1.56 ± 0.09	0.33 ± 0.05^c^	6.0	0.46 ± 0.07^e^

^a^PD; periodontal pocket depth, PI; plaque index, GI; gingival index, BOP; bleeding on probing.

^b-e^Statistical differences between two groups revealed by Student's t-test between two groups (^b, c^*P *< 0.05 and ^d, e^*P *< 0.001).

### Relationships of developmental disabilities and clinical parameters with detection of periodontopathic bacterial species

**Figure 3:**
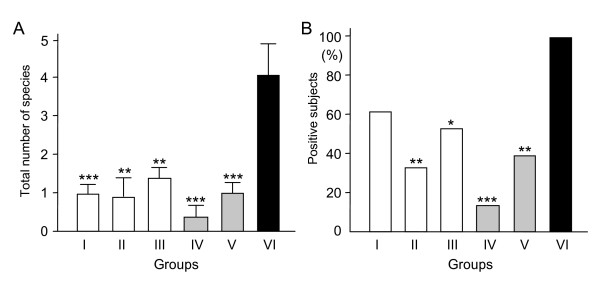
**Correlation of detection of periodontopathic species with general conditions**. Average number of the 10 tested species (A) and rate of subjects with at least 1 of those species (B). Group I, mental retardation (MR) (n = 60); Group II, cerebral palsy (CP) (n = 16); Group III, autism (AU) (n = 28); Group IV, MR/CP, (concomitant MR and CP) (n = 17); Group V, concomitant MR/AU (n = 32); Group VI, concomitant MR/CP/AU (n = 7). Statistical analyses were performed by ANOVA (Tukey's Multiple Comparison Test) (A) and Fisher's exact probability test (B). * *P *< 0.05, **P < 0.01, ***P < 0.001.

**Figure 4:**
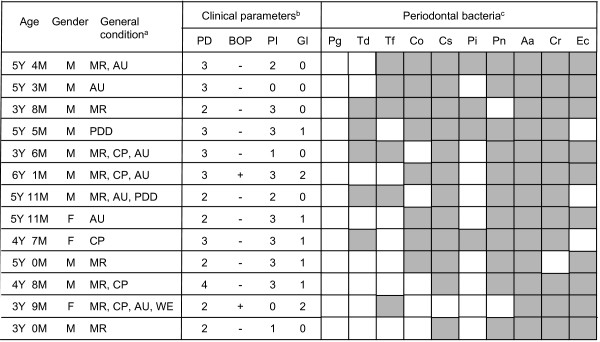
**Summary of subjects with large numbers of the test species detected**. ^a^MR, mental retardation; AU, autism; PDD, pervasive developmental disorders; CP. cerebral palsy; WS, West syndrome. ^b^PD, periodontal pocket depth; BOP, bleeding on probing; PI, plaque index; GI, gingival index, ^c^Closed squares indicate the presence of at least one of the following bacterial species: Pg, *Porphyromonas gingivalis*; Td, *Treponema denticola*; Tf, *Tannerella forsythia*; Co, *Capnocytophaga ochracea*; Cs, *Capnocytophaga sputigena*; Pi, *Prevotella intermedia*; Pn, *Prevotella nigrescence*; Aa, *Aggregatibacter actinomycetemcomitans*; Cr, *Campylobacter rectus*; Ec, *Eikenella corrodens*.

The 187 subjects were divided into 6 groups; MR only (Group I; n = 51), CP only (Group II; n = 16), AU or PDD only (Group III; n = 40), both MR and CP (Group IV; n = 17), both MR and AU or PDD (Group V; n = 41), and MR, CP and AU (Group VI; n = 7). The values for PD and PI were not significantly different among these groups, whereas the value for GI in Group VI was significantly higher than that in Groups I, III, IV, and V (*P *< 0.01) (Table 4). In addition, the rate of BOP in Group VI was significantly higher than that in Groups I (*P *< 0.01) and V (*P *< 0.05). The total number of species in Group VI was 4.14, which was significantly greater than that in the other groups (*P *< 0.01) (Figure 3A). Furthermore, all subjects were positive for at least 1 species in Group VI, which was greater than in the other groups (Figure 3B). The distribution rates of RC species and Cr in Group IV were 61.5% and 91.3%, respectively, which were significantly higher than in other subjects with 4 or fewer species detected (*P *< 0.001). A summary of subjects with more than 5 species detected is provided in Figure 4. Approximately half of all subjects were complicated with multiple developmental disabilities.

**Table 4:** Periodontal conditions of subjects based on diagnosis of general condition

**Groups^a^**	**Number of subjects**			**Clinical parameters^b^**			
		
	**Boys**	**Girls**	**Total**	**PD** **[Mean ± SE (mm)]**	**PI** **[Mean ± SE]**	**GI** **[Mean ± SE]**	**BOP** **-positive** **(%)**
I. MR only	33	18	51	2.29 ± 0.10	1.80 ± 0.16	0.35 ± 0.07	0
II. CP only	10	6	16	2.50 ± 0.16	2.07 ± 0.23	0.50 ± 0.18	12.5
III. AU or PDD only	33	7	40	2.38 ± 0.11	1.48 ± 0.19	0.38 ± 0.09	10.0
IV. MR & CP	8	9	17	2.29 ± 0.19	1.12 ± 0.26	0.24 ± 0.14	5.9
V. MR & AU or PDD	35	6	41	2.32 ± 0.10	1.46 ± 0.16	0.29 ± 0.11	7.5
VI. MR, CP & AU	6	1	7	2.43 ± 0.20	2.14 ± 0.46	1.29 ± 0.29^c^	42.9^d^

^a^MR, mental retardation; CP, cerebral palsy; AU, autism; PDD, pervasive developmental disorders.

^b^PD; periodontal pocket depth, PI; plaque index, GI; gingival index, BOP; bleeding on probing.

^c^*P *< 0.01 vs MR only, AU or PDD only, MR & CP and MR & AU or PDD groups (ANOVA).

^d^*P *= 0.0011 vs MR only, *P *= 0.3047 vs CP only, *P *= 0.0566 vs AU or PDD, *P *= 0.0593 vs MR & CP, and *P *= 0.0328 vs MR & AU or PDD groups (Fisher's exact probability test).

## Discussion

A great number of studies performed worldwide have reported the prevalence of periodontopathic bacterial species in dental plaque specimens [2-8,14]. Among the 10 species analyzed in the present study, the detection rates of *P. gingivalis*, *P. intermedia*, *T. denticola*, and *T. foysythia *are considered to be extremely low. Analysis of periodontally healthy children aged 5-10 years in Finland showed that the detection rate for *P. gingivalis *was 5% [19], while another study performed in Greece that analyzed children aged 4 or 5 years old reported detection rates for *P. gingivalis *and *T. forsythia *of 2.6% and 7.5%, respectively [20]. In addition, a previous analysis of 20 healthy Japanese children aged 2-12 years old did not find any subjects positive for *P. intermedia *or *T. denticola *[21]. In the present study, none of the subjects were positive for *P. gingivalis*, while the detection rates for *P. intermedia*, *T. denticola*, and *T. foysythia *were each less than 5%. These species are not considered to be common periodontopathic bacteria in early childhood, except when advantageous conditions for their colonization are present. On the other hand, another study reported that the detection rates for 6 other species, *P. nigrescence*, *C. rectus*, *E. corrodens*, *A. actinomycetemcomitans*, *C. ochracea*, and *C. sputigena*, in periodontally healthy Japanese children were comparatively high [8]. As for those in children examined in the USA, analysis of 114 subjects revealed that the detection rates of *A. actinomycetemcomitans*, *C. ochracea*, *C. rectus*, and *E. corrodens *ranged from approximately 30-50% [22], which are similar with those reported for periodontally healthy Japanese subjects. The present study also revealed that the detection rates of these species gradually increased with age, indicating that these species are early colonizers.

It is well known that individuals with Down's syndrome, one of the most common causes of mental disabilities in children, are susceptible to early onset of periodontitis due to impaired immune response, fragile periodontal tissue, and early senescence [23,24]. In a previous study that detected periodontopathic bacterial species in children with Down's syndrome using the same methods as in the present study, large numbers of species were detected in dental plaque specimens [16]. On the other hand, a comparison of the bacterial profiles of adult subjects with Down's syndrome with those of adult subjects with mental retardation could not find any differences [25], indicating that the early onset of periodontitis identified in Down's syndrome may not be derived from only the presence of periodontopathic bacteria. In the present study, 11 subjects had Down's syndrome, and their clinical parameters and numbers of periodontopathic bacteria detected were not significantly different as compared to subjects with other types of developmental disabilities [Table 1, see Additional file 2]. It is possible that these findings reflected the low age of the subjects, and/or that the guardians and daycare center staff successfully maintained the oral hygiene of the subjects.

To our knowledge, there is a limited number of reports dealing with gingival conditions of subjects with developmental disabilities, such as mental retardation, cerebral palsy, and autism, who require special care for maintaining oral hygiene and receiving dental treatment [26,27]. Molecular biological detection of periodontopathic species in dental plaque specimens collected from a large number of such subjects was performed in the present study. It should be noted that 70% of all our subjects were males and that this gender bias may have affected the results, though the effect of gender difference on the prevalence of periodontopathic species is controversial. In the present subjects, the total number of the 10 species in males (n = 136) was 1.18 ± 0.17 species, which was nearly the same as that in females (1.08 ± 0.23) (n = 51). Since the distribution of the subjects in each age group was nearly the same for both genders, we consider that gender differences had no significant effects on the detection rates of periodontopathic species in the present study.

Previous results have shown that a considerably higher frequency of inflamed gingival surfaces and pathological gingival pockets are present in children with severe mental retardation as compared to healthy children in spite of similar frequencies of dental care, which has been speculated to be caused by a lack of cooperation by the patient during treatment [28]. We performed the present study as a part of a program of annual periodical dental examinations requested by the daycare centers. Thus, only simple evaluations of periodontal conditions were done in order to focus on the screening of children with severe periodontal conditions. It is fortunate that none of the subjects analyzed in the present study had extremely poor gingival conditions, possibly due to their younger ages. In addition, we were surprised to find that the mean values for the total number of the tested 10 species ranged from 0.38 for subjects aged 1-2 years old to 1.98 for those aged 5-6, which were much lower as compared to systemically healthy children who previously attended our clinic and were analyzed (2.17 for 2-year-old subjects, 3.00 for 6-year-olds) [7]. It should be noted that the same protocol was used for detection of the 10 periodontopathic bacterial species with the same chemicals obtained from the same manufacturers in the present and previous studies. Thus, we consider that the daycare center staff and guardians of the children likely paid particular attention to maintain the oral hygiene of the subjects.

In spite of the low overall detection rates for the tested periodontopathic bacterial species, several subjects were positive for a large number of species. In general, early detection of and treatment for periodontal diseases are considered to be important, whereas the best approach to managing them is believed to be prevention [1]. Identification of subjects at a high risk for periodontitis is useful using a technique based on species detected in dental plaque. In order to construct a method to determine such high risk subjects, we previously compared the distribution of periodontopathic species and gingival conditions in the same subjects over a 7-year period, and found that the detection rate of the red complex species after 7 years was significantly higher in subjects positive for 2 or more species as compared to those with 1 or 0 detected (odds ratio 17.5) [14]. Among the present subjects, the ratios of subjects positive for more than 2 species in groups aged 1-2, 3, 4, and 5-6 years old were 14%, 35%, 16%, and 42%, respectively, which indicates that approximately one-third of all the present subjects could be categorized as high risk, even though the rates were quite low as compared to those for systemically healthy children analyzed in our previous study [7]. We intend to continue to perform careful follow-up examinations to observe the transitional changes of gingival conditions in these subjects.

In addition to the total number of species detected, identification of those specifically associated with the onset of periodontitis is considered to be important. In studies of Japanese children and adolescents, we have been focusing on the red complex species and *C. rectus *[10,11,14]. Prior to the establishment of annual dental examinations at daycare centers for disabled children, we devised the protocol used in the present study by considering various convenient approaches to be utilized with the subjects and gained beneficial results to indicate high risk subjects based on the results of previous studies. Molecular biological analyses revealed that approximately 5% of all the present subjects possessed the red complex species (*T. denticola *and/or *T. forsythia*), while 20% possessed *C. rectus*. We think that individuals possessing 1 or more of these species should be considered at high risk for the onset of periodontitis. In addition, it is important to compare the results of periodontopathic bacterial detection in dental plaque samples obtained at annual dental examinations.

On the other hand, it is better to analyze saliva samples as compared to dental plaque samples, as the former reflect the whole oral cavity, while the latter only represent a localized area [15]. However, collection of dental plaque samples was considered more convenient for the present study, due to the limitations of our developmentally disabled patients. Hayashi et al. [29] analyzed Japanese children aged 4-6 years old and recommended that 2 or more random sites for *C. rectus *and 7 or more sites for *T. forsythia *should be sampled in order to specify subjects positive for those species. Thus, it is possible that additional subjects in the present study were positive for *C. rectus *or *T. forsythia*. Modification of the procedure for sample collection from a single tooth to multiple teeth should be considered for future studies.

Analysis of data based on the classification of subjects with MR only, CP only, and AU or PDD only did not reveal significant differences in regard to the clinical parameters or for detection of periodontopathic species in dental plaque. In contrast, the group of the subjects with concomitant MR, CP, and AU had greater values for GI and BOP-positive rate. It is reasonable to speculate that individuals with multiple developmental disabilities have great difficulties with maintaining oral health and undergoing dental treatment as compared to subjects with a single disability. Significantly greater numbers of periodontopathic species were identified in the group of subjects with concomitant MR, CP, and AU, which led us to conclude that special approaches should be considered for such subjects to prevent worsening of their gingival conditions.

In summary, we performed clinical examinations and used a molecular biological method for detection of periodontopathic bacterial species in a large numbers of patients with developmental disabilities. We designated subjects at high risk for worsening gingival conditions based on the total number of bacterial species detected and identification of specific high risk species previously reported. It will be important to perform continuous follow-up examinations for those designated as high risk, as well as to encourage daily oral care by staff and/or guardians, periodical check-ups by dentists, and, if necessary, early professional intervention.

## Conclusion

We analyzed periodontal conditions and performed detection of 10 periodontopathic bacterial species in dental plaque samples obtained from 187 Japanese children with developmental disabilities aged 6 years old and younger. Approximately one-fourth of the present subjects with disabilities who possessed at least 1 of *T. denticola*, *T. forsythia*, and *C. rectus *were at possible risk for periodontitis. Follow-up examinations as well as preventive approaches will be important for those patients.

## Competing interests

The authors declare that they have no competing interests.

## Authors' contributions

The entire study protocol was constructed by KN and MM under the supervision of TO. Clinical examinations were performed by MM, and collection of specimens was done by RO and KF. RN designed the protocol for molecular biological detection of the periodontopathic bacteria, which was carried out by SN, AY, AK, and HN. Statistical analyses and interpretation of the results were performed by SN and AY. KN wrote the manuscript, under the supervision of TO. All authors read and approved the final manuscript.

## Pre-publication history

The pre-publication history for this paper can be accessed here:


